# Optimization of Optical Phase Profile in Beam Deflector with Advanced Simulation Method for High Diffraction Efficiency

**DOI:** 10.3390/mi13050802

**Published:** 2022-05-21

**Authors:** Andrey Manko, Young Kim, Aleksander Morozov, Serguei Palto, Kanghee Won, Hong-Seok Lee

**Affiliations:** 1 Samsung R&D Institute Russia, Office 1500, 12 Bldg.1, Dvintsev Str., 127018 Moscow, Russia; a.manko@samsung.com (A.M.); alexander.morozov@samsung.com (A.M.); 2 Advanced Sensor Lab, Device Research Center, Samsung Advanced Institute of Technology, Suwon 437-826, Korea; onyas.kim@samsung.com; 3 P.N. Lebedev Physical Institute of the Russian Academy of Sciences, 53, Leninskiy Prospect, 119991 Moscow, Russia; 4 Shubnikov Institute of Crystallography, Federal Scientific Research Centre “Crystallography and Photonics”, Russian Academy of Sciences, 119333 Moscow, Russia; serguei.palto@gmail.com; 5 Department of Electrical and Computer Engineering, Seoul National University, Seoul 151-742, Korea; lhs12100@snu.ac.kr

**Keywords:** beam deflector, nematic liquid crystal, diffraction efficiency, optical phase control

## Abstract

Controlling the phase of light with a high efficiency and precision is essential for applications in imaging, tunable devices, and optical systems. Spatial light modulators (SLMs) based on liquid crystals (LCs) have been regarded as one of the best choices for the generation of phase profiles for the steering of light. The upper glass substrate has an unpatterned electrode for a common electrode, while the lower glass substrate has one-dimensional micro-patterned electrodes for controlling the single pixel level by the applied voltages. By applying different voltages to each electrode to create a sawtooth-shaped phase profile, the collimated input beam is deflected to the desired angle. To maximize the diffraction efficiency (DE) values, an advanced simulation method has been developed to find the optimized phase profile through the analysis of LC director distributions. The resulting diffraction patterns are investigated both computationally and experimentally, with a good agreement between the results obtained. Finally, the beam deflector (BD) system with an advanced driving algorithm has a high 1st order DE, about 60%, 37%, and 7.5% at 1°, 2.5°, and a maximum steering angle of 7.5°, respectively. The LC director distributions in relation to various diffraction angles are simulated and an experimental success in realizing enhanced DE for the beam steering device is presented.

## 1. Introduction

Nonmechanical types of beam steering systems without moving parts have many advantages compared to mechanical systems, and they can be a good choice for various applications such as diffractive lens systems [1], optical communications [2], phased array beam steering systems [3], waveguide-based electro-optics [4], and so on.

Liquid crystal (LC) has been widely used in various applications such as tunable lenses [5,6], holographic displays [7,8,9], smart glasses [10], random lasing [11], and intensity modulators [12] by taking advantage of a low cost with a relatively simple process, and it has also been used for beam steering as an electro optical modulators [13,14,15]. Among them, nematic liquid crystals (NLCs) have the advantage of a low driving voltage, robust manufacturing process, and a low cost; in particular, the NLCs with a high birefringence are of special interest because they can reduce switching time with a small cell gap [16,17,18]. For this reason, we optimized new synthetic methods to obtain liquid-crystalline laterally substituted polyaromatic derivatives of long rigid molecules, such as *n-*quaterphenyl and *n-*quinquiphenyl. In this work, substituted cyclic fragments are formed via new modifications of well-known condensation reactions. The new synthetic methods use readily available initial components and are characterized by simplicity. Finally, the NLC mixture named HBM-2 with high birefringence was developed [19].

A gradient phase profile is generated within an LC layer when an external electric field is applied to the upper and lower electrodes. The LC directors with positive dielectrics rotate along the electric field, and the light experiences different phase retardations with the distribution profiles of the tilt angles. A blazed diffraction grating can be created from a sawtooth phase profile, and the input beam is then steered to the desired angle. The BDs with large diffraction angles and high angular resolutions were developed by achieving a fine pitch at the micro-scale [20]. However, diffraction efficiency (DE) decreases drastically as the diffraction angle increases due to the fringing field effect occurring between adjacent micro-scale patterned indium-tin oxide (ITO) electrodes on the lower glass substrate.

The main purpose of this study is to improve the DE through the optimization of LC materials and driving algorithms. The cell gap is reduced by using a high refractive NLC mixture of HBM-2 as mentioned above. However, the most important way to maximize the DE is to calculate the actual optical phase profile from the fine tuning in the simulation and fit it as closely as possible to the ideal phase profile. This advanced simulation method requires an accurate prediction of the LC directors’ distribution, which is made possible by building a complete numerical model of the LC layer. The numerical model obtained by setting input voltages for each electrode and LC parameters is then converted into an optical phase profile. Finally, the intensity of the diffraction orders and beam focusing from the optical phase profile is accurately predicted using fast Fourier transform (FFT) and inverse FFT techniques.

## 2. Materials and Methods

### 2.1. Working Principle

#### Steering Angle and Spatial Resolution

**Figure 1:**
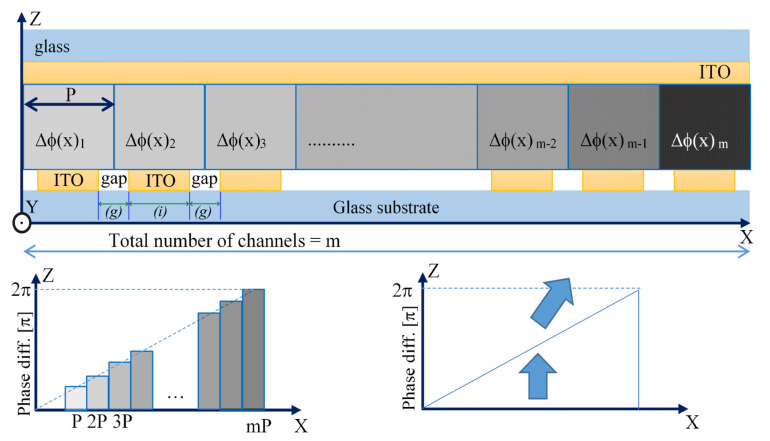
LC-based BD cell geometry and concept. The fill factor (FF) of the grating is the ratio of the ITO electrode width (*i*) to the gap (*g*) on the lower substrate. In this example, FF = *i/g* = 1.5/0.5 μm. The full period (*p*) of the grating in the example shown is 2 μm.

Figure 1 depicts the structure of a BD with ITO electrodes and LCs for the birefringence changes. The optical phase profile is created by the variable effective refractive index of the LCs sandwiched between the upper and the lower substrates. Applying the different voltages to the ITO electrodes on the lower substrate, we can observe the difference in the effective refractive indices of the LC medium between them. Each patterned ITO electrode on the lower substrate can be driven independently by the external voltage from the BD driving module. The driver IC has a total of 360 channels, and the maximum driving voltage of a driver IC is ±9 V. The BD driver module has two ICs for a total of 720 channels. An optical deflection caused by changing the effective refractive index of the LCs is similar to that occurring in a real prism, deflecting the transverse magnetic (TM) wave falling in the XZ-plane. We designed the BD system so that the refractive index difference Δ*n*, and the cell gap *d*, can both provide a phase modulation larger than 2π to satisfy the phase matching condition. We are interested in the phase modulation above 2π for all three wavelengths of 460 nm, 520 nm, and 638 nm. A polyimide was coated onto the upper and lower substrates to create a homogeneous planar LC alignment that yields a low pretilt angle, and the minimum thickness *d* = 2.5 μm is enough to produce the required phase retardation above 2π even at the longest wavelength of 638 nm. A minimum basis of 2π phase shift was applied to create a sawtooth phase profile because all 2πn phase shifts are equivalent from a phase point of view, as shown in Figure 1.

As described in Equation (1), both the maximum and minimum steering angles *θ* are determined by the number of electrodes in one-unit prism and the total number of channels.

(1) $$\Theta =arcsin\left(\frac{\lambda }{n\times p}\right),\;n=\frac{m}{i}\;(0<i\;\leq \;360)$$

where *λ* is the wavelength of the light, *n* is the total channel number for a unit prism that is defined by *m*/*i*, *m* is the total number of channels, and *i* is the total number of unit prisms. Pixel pitch (*p*) is the sum of the electrode width (*i*) and the gap (*g*). 

For instance, the pixel pitch *p* is equal to 2 μm and the total channel number, *m*, is decided as 720 according to the number of drive ICs. According to Equation (1), the maximum steering angle when the total number of unit prisms, *i*, is 360 is *θ_max.B_* = 6.604° for *λ* = 460 nm. For other wavelengths, the maximum steering angle is larger: *θ_max.G_* = 7.470° for *λ* = 520 nm, and *θ_max.R_* = 9.178° for *λ* = 638 nm. The angular resolution is defined by the smallest angle available when the *i* = 1. The resolution values are 0.018°, 0.021°, and 0.025° for the wavelengths of 460 nm, 520 nm, and 638 nm, respectively.

### 2.2. Advanced Simulation Method

We used the LCD TDK software to predict the three-dimensional LC director distributions. The program uses the finite-element method for the minimization of free energy of an inhomogeneous LC bulk within the frames of the Oseen-Frank elastic model [21,22]. In all cases, an infinite anchoring energy assumption was applied. In our LCD TDK simulation, a three-dimensional mesh of finite volume elements for *dx* = *dy* = *dz* = 0.1 μm was used to calculate all three components (*N_X_*, *N_Y_*, and *N_Z_*) of the LC director $\vec{N}$.

**Figure 2:**
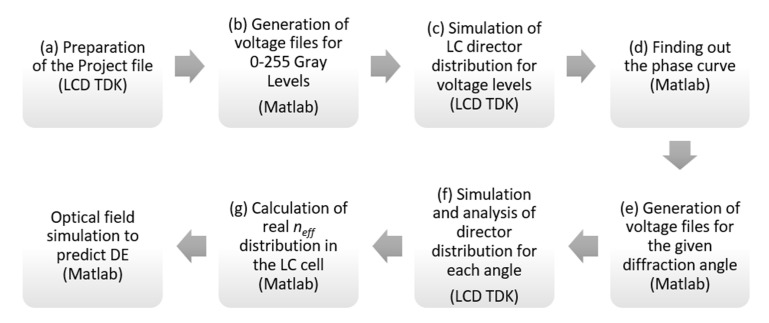
Simulation workflow for the optimization of the phase profile in the BD.s.

LCD TDK software tool served as the computational backend for MATLAB. Simulation workflow includes several steps aimed at the optimization of DE in our LC-based BD cell is shown in Figure 2. We describe the details for each step below.

#### 2.2.1. Preparation of the LCD TDK Project File

**Figure 3:**
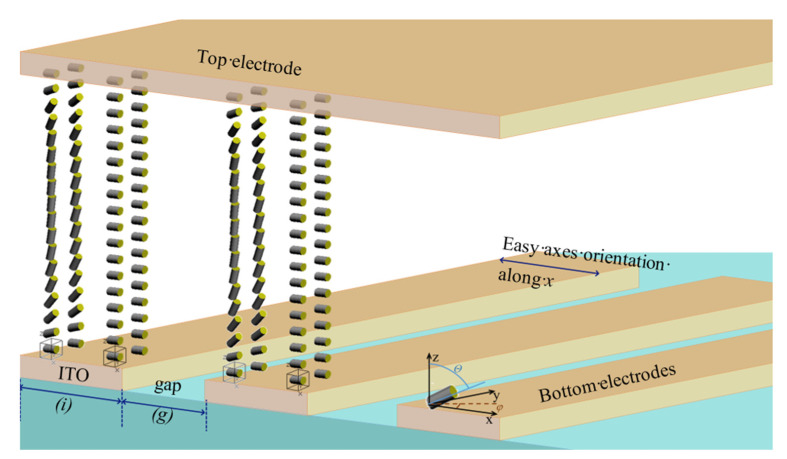
Visualization of the modeling results of LC molecules for generation of a blazed grating in the LC cell with a thickness of 2.5 μm. The initial orientation of the LC molecular was set to the *x*-axis which is perpendicular to the electrodes.

Initial boundary conditions for the simulation of the LC director distributions in the layer were set to closely match the real BD cell, including the height of ITO electrodes and alignment layers, pre-tilt angle of LC molecules adjacent to the electrodes, anchoring of LC molecules to the substrate, and so on. The boundary conditions, electrode structure, elastic constants, and refractive indices for our LC materials were set using MATLAB scripts. Since the liquid crystal is sensitive to temperature, its parameters and electrical properties also change accordingly [23,24]. To match the conditions of the simulation and the experiment, the parameters of the liquid crystal measured at room temperature shown in Table 1 were inserted into the simulation and the DE experiment of the BD was also tested at the same temperature. As shown in Figure 3, an irregular quadrilateral surface that is periodic in one direction and the position of the points in space are given by their Cartesian coordinates. The *x*-axis is the direction of the periodicity, and the *y*-axis is parallel to the electrodes. The incident light lies in an XZ-plane which is perpendicular to the grooves.

#### 2.2.2. Generation of Files for Each Voltage Level

The structure of the BD described above is used to generate 256 files with voltage levels between 0 and 9 volts, using the linear law U = (9/255) × GL, where GL is the gray level value ranging from 0 to 255. MATLAB script is used at this step to generate the set of project files for LCD TDK, by applying different voltage levels within the same geometry.

#### 2.2.3. LCD TDK Simulation to Find out LC Director Distribution for Each Gray Level

At this stage, we run an independent simulation for each electrode. Each voltage level produces different LC director distributions between electrodes in the bulk. Using the LCD TDK in-built ability, we save the three-dimensional LC director distribution into a binary file. Afterwards, we parse this file and analyze it using MATLAB to find out the phase retardation level on the top XZ-plane for each gray level value. The phase retardation is determined by the summation of optical path values in each finite element with *dx = dy = dz* size, along the *z*-coordinate.

#### 2.2.4. Determination of the Phase Curve

**Figure 4:**
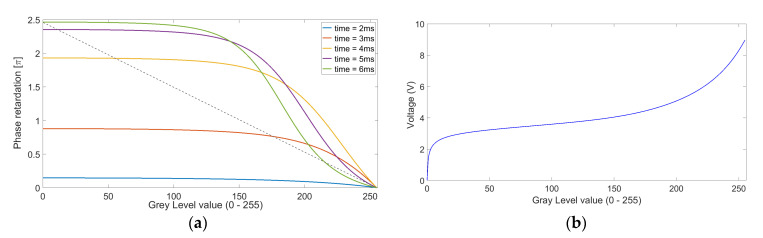
(**a**) Phase retardation values as a function of gray level values. The given case is for HBM-2 material (LC layer thickness *d* = 2.5 μm, i/g = 1.5/0.5 μm, light source wavelength *λ* = 520 nm). The linear dashed line indicates the voltage calibration corresponding to linear phase change. (**b**) DC voltages as a function of gray level values corresponding to a linear phase change.

At this step, we determine the dependence of the phase on time, finding the level of phase modulation that reaches a certain saturation level above 2π. The shorter times will result in unsuitable phase modulation levels below 2π, so the maximum simulation time of several milliseconds is then fixed. The typical simulation time can be as low as six milliseconds, as shown in Figure 4a.

To obtain the blazed phase profile, MATLAB is used to derive the voltage profile at the second step. A phase–voltage curve for the given LC layer thickness is generated at each wavelength. This dependence, or phase curve, is then used to determine the calibrated DC voltage controller gray levels in 0 to 255 scale so that the desired phase change becomes linear. Voltages as a function of gray level information is stored in the controller’s memory, as shown in Figure 4b.

#### 2.2.5. Generation of Voltage Files Distribution for the Given Diffraction Angle

We then use MATLAB to generate a phase profile for the desired diffraction angle. For example, to obtain a diffraction angle of 1.524° at a wavelength of 520 nm, one-unit prism of a blazed geometry was created within 10 channels, then 72 identical unit prisms are created through a total of 720 channels at a wavelength of 520 nm. Using the phase curve mentioned above, the given geometry translates to the LCD TDK project file with the predetermined voltage distribution between the electrodes. This is the simulation stage, where we begin constructing the full phase array of electrodes for beam steering. In MATLAB, the DC voltages as a function of gray levels can be set to any analytical form. Therefore, the real voltage distribution over the array of electrodes can be also set independently.

#### 2.2.6. Simulation and Analysis of LC Director Distribution for each Diffraction Angle

Next, for the pre-determined voltage distribution, we compute the LC director distributions in three-dimensional blazed-grating geometry. Again, we perform an independent LCD TDK simulation for each blazed profile which correspond to certain diffraction angles. 

#### 2.2.7. Calculation of the Effective Refractive Index Distribution in the LC Cell

In the case of our geometry shown in Figure 1, we are mainly interested in the propagation of a TM wave through the XZ-plane. The two-dimensional distribution of LC directors in each of *n* sub-volumes in the XZ-plane (each thickness *dy* = 0.1 μm) repeats itself. That is reason to use averaged *N_Y_* values considered with only two *N_X_*, *N_Z_* components of the director. Hence, the spatial distribution of the average refractive index in this plane is given by the equation.

(2) $$n\left(x,z\right)=\frac{n_{o}n_{e}}{\sqrt{\left[n_{o}^{2}N_{x}^{2}\left(x,z\right)+n_{e}^{2}N_{z}^{2}\left(x,z\right)\right]}}$$

where *n_o_* is the ordinary refractive index and *n_e_* is the extraordinary refractive index of the LC mixtures. The spatial modulation of the effective refractive index in the x-direction for the TM wave, propagating along the z-coordinate, is defined by:

(3) $$\Delta n\left(x\right)=\frac{1}{d}\int_{0}^{d}\left[n_{e}-n\left(x,z\right)\right]dz$$

where *d* is the thickness of the LC layer. The phase modulation in the inhomogeneous static cell for the given cell gap (*d*) and wavelength (*λ*) can then be expressed as:

(4) Δφ(x)=2πΔnd(x)/λ

Hence, after the calculation of the effective refractive index distribution Δ*n*(*x*) in the LC cell, we obtain the phase modulation profile for the XZ-plane along the coordinate x for the whole array of electrodes. Each calculated phase level along the x-axis is derived from the spatial distribution of the director with the given accuracy of the simulation.

The LC directors of the BD cell are extracted, on which the distribution of the LC directors was calculated based on the Ericksen-Leslie equation [25,26].

(5) $$\gamma n_{i}-\frac{\partial F}{\partial n_{i}}-\gamma_{1}N_{i}-\gamma_{2}n_{j}A_{ji}+{\left(\frac{\partial F}{\partial n_{i,j}}\right)}_{,j}=0$$

where the *F* is free energy, *γ* is the Lagrange multiplier, and *γ*_1_ is viscosity torque.

**Figure 5:**
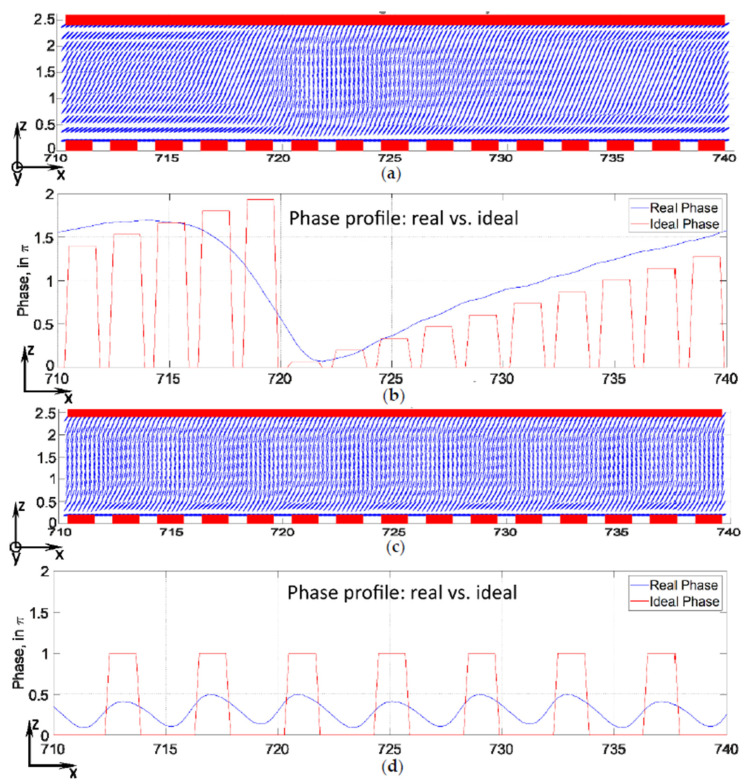
Visualization of simulation results for a blazed grating formed in the LC cell: (**a**) distribution of LC directors (in XZ-plane, cross-section) in the “on-state”, when each electrode has reached its target voltage level, at a diffraction angle of 1.5°; (**b**) real (curves) and ideal phase profiles (columns) at the same length, at a diffraction angle of 1.5°; (**c**) distribution of LC director (in XZ-plane, cross-section) in the “on-state”, when each electrode has reached its target voltage level, at a diffraction angle of 7.47°; (**d**) real (curves) and ideal phase profiles (columns) at the same length, at a diffraction angle of 7.47°.

The boundary condition of the upper ITO electrode is set to ground (0 V), while the lower patterned ITO electrodes are set to sweep mode voltage, which changes the voltage from 0 to 9 V. As mentioned earlier, the upper and lower glass substrates are coated with low pretilt polyimide for the real BD cell, the tilt angle and the azimuthal angle (*θ* = 86°, *φ* = 0°) near the substrates are defined for the initial distribution of LC director in the simulation. In the field off state, the LC directors are horizontally aligned, while the LC directors rotate at different values of potential differences from 0 to 9 V. As shown in Figure 5a,c, the simulated results show that the tilt angles in the BD cell drastically change at the mid-range of the LC layer because the molecules are rotated by the electric field as it is far away from the anchoring force around the substrate. Figure 5b,d indicate the voltage-dependent phase retardation caused by the change in the birefringence of the LC. The ideal phase value is indicated by a column, and the actual phase value is indicated by a curve.

## 3. Results

A DE has been studied by using MATLAB and LCD TDK as well as experiments. As mentioned earlier, a blazed profile is employed to maximize DE and it varies according to the diffraction angles. The DE of the *m*^th^ order is given by [27]:

(6) $$\eta_{m}={\left|b_{m}\right|}^{2}$$

(7) $$b_{m}=\frac{1}{\Lambda }\int_{0}^{\Lambda }exp\left[i\Delta \varphi \left(x\right)\right]exp\left(-i\frac{2\pi mx}{\Lambda }\right)dx,$$

where *m* is the diffraction order (*m* = 0, ±1, ±2, …), *Λ* is the grating period, and Δ*φ(x)* is the phase modulation across the grating. 

For the calculation of the DE, the sum of all the diffraction orders is mostly used, and it is calculated using the equation as follows [28]:

DE = (*I*−*I*_0_)/*I* (8)

where *I*_0_ is the intensity of zero order consisting of undiffracted light and *I* is the intensity of the incident laser beam.

However, since the holographic display system uses only first-order diffracted light, the DE in this study was calculated only for first-order diffraction using the following formula:

DE = *I*_str_/*I*_off_ (9)

where *I_str_* is the intensity of only the first-order diffraction and *I_off_* is the intensity of the incident laser beam penetrating the LC-based BD.

### 3.1. Optical Field Simulation to Predict Diffraction Efficiency

We implemented algorithms of a free space propagation model based on the FFT in MATLAB for the stack of optical elements. To avoid direct evaluation of the Fresnel-Kirchoff integral for the propagation of the optical wavefront from a plane, an alternative evaluation algorithm was suggested. The sampled version of the integral was used, and it was calculated by means of the FFT. First, an FFT of the input signal was multiplied by the sampled version of a quadratic phase factor, and then an inverse FFT of that product was taken to calculate the proper sampled version’s expression for the diffraction pattern.

**Figure 6:**
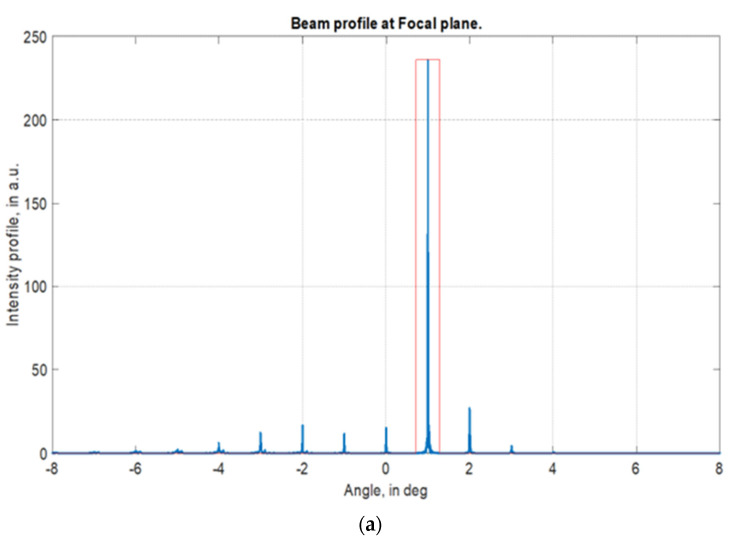

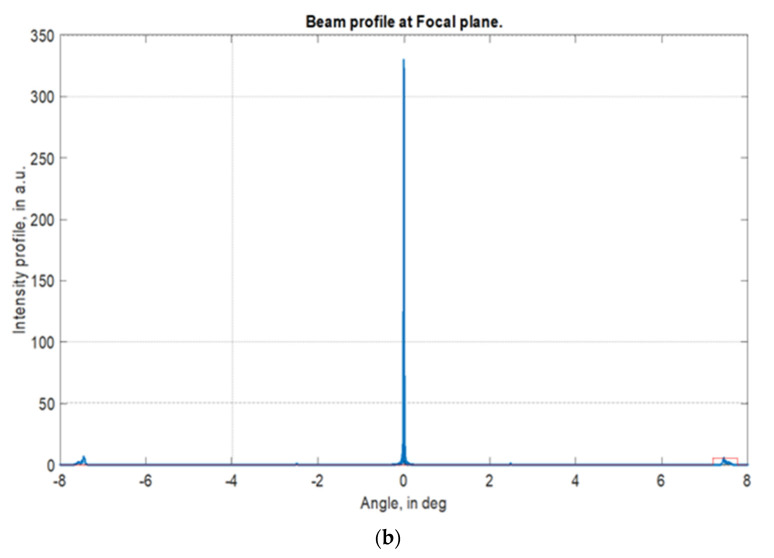
Beam profiles and DE values calculated for the full optical field. Simulation of the LC cell gap *d* = 2.5 μm and the wavelength of the light source is *λ* = 520 nm. (**a**) Calculated first order DE is 61.5% at a diffraction angle of 1.5°; (**b**) calculated first order DE is 6.4% at a diffraction angle of 7.47°.

Similar to our experimental setup, the LC-based BD cell was stacked with an ideal lens having a back focal distance of several tens of millimeters. The beam distribution at the focal plane accounted for intensities of all diffraction orders. However, the beam profile of operating at the first order of diffraction was registered within the “eye pupil” equal to tens of millimeters. This method allowed us to evaluate near field propagation (i.e., 50–200 mm) and the calculated DE at diffraction angles of 1.5° and 7.47° are shown in Figure 6.

### 3.2. Experimental Measurement of Diffraction Efficiency

**Figure 7:**
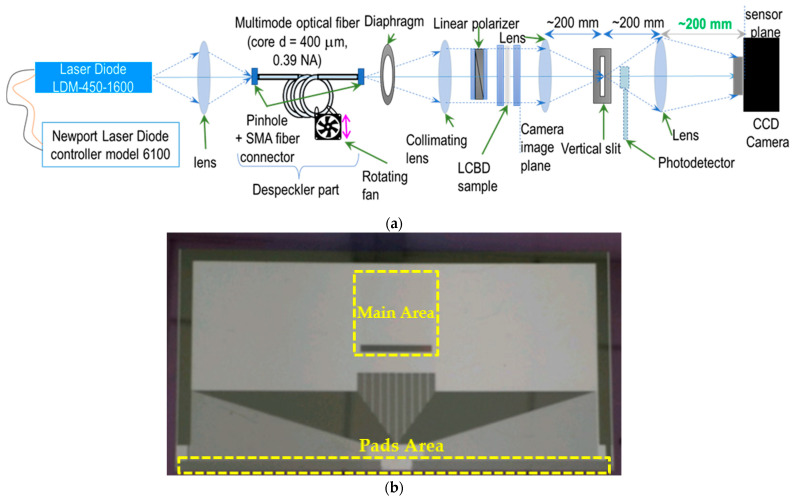
(**a**) The schematic diagram of components for DE measurement; (**b**) actual BD cell showing the main area containing ITO-based patterned electrodes with high birefringence of NCL and the pads area for 720-channel driver IC.

An experimental setup was constructed and an LC-based BD cell is employed to measure DE values. Figure 7 illustrates the experimental setup for the DE measurement. The laser diode light source, optical fiber despeckler, collimating system, and the vertical slit that allows only the first diffraction order to pass were used. Additionally, the lens with a back focal distance of 10 mm was stacked with the LC-based BD cell, and the intensity of the beam profile at this focal distance was registered within the spot (“eye pupil”) equal to 0.1 mm.

Alternating current voltages provided by an arbitrary function generator were then applied across the electrodes. The upper bare ITO electrodes were connected to the ground, while different voltages were applied to the lower patterned ITO electrodes. The experimental DE value was determined to be the ratio of the photocurrent intensity for the first-order diffraction to that of the zero-order beam, according to Equation (9).

**Figure 8:**
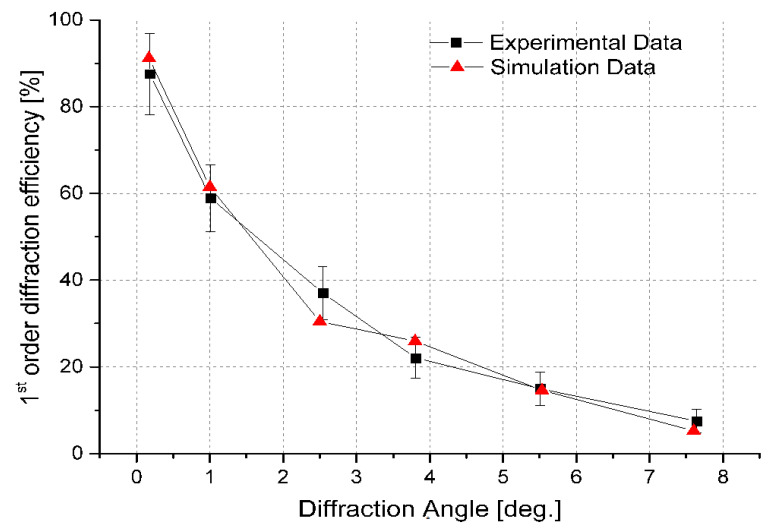
Experimentally and simulated DE of a LC-based BD cell as a function of the diffraction angle. The square and triangle denote the experimental and simulation data, respectively. The error bars are used to indicate the errors which are calculated by dividing the standard deviation by the square root of the number of experiment measurements.

The results obtained from the simulation and the experiment involving the DE have been reiterated in Figure 8 for a direct comparison, and six different points from the minimum to the maximum diffraction angles were plotted. As described in Equation (9), DE is defined as the ratio between the intensity of the first diffracted order and the total intensity in the absence of an applied external voltage. The DE curves demonstrate that the values start high at a low diffraction angle, however, the values are decreased as the diffraction angle increases. The errors were calculated by dividing the standard deviation by the square root of the number of experiment measurements, and a very good agreement is obtained between simulated and experimental results.

## 4. Conclusions

In conclusion, an electrically tunable transmission-type of BD that consists of ITO-based patterned electrodes with high birefringence of NLC is proposed, and an analysis of the LC directors and DE is presented. The DE is calculated using FFT analysis based on the LC director distributions and is compared to the experimental results to validate the model. An excellent consistency with those results is obtained between calculations and measurements.

To enhance the DE, the LC directors were fine-tuned by applying an additional driving algorithm, such as a gamma function, and the actual phase profile was closely matched to the ideal phase profile. In particular, the simulation results confirmed that the fringing field effect became more severe due to the interference of adjacent electrodes at a high diffraction angle, which resulted in a decrease in DE. To optimize this, a special phase profile of 0-π-0-π was applied to the maximum diffraction angle.

This work demonstrates how to effectively optimize the optical phase profile in BD using a numerical model, and this advanced simulation method can be widely used for many types of beam steering devices using LC with the advantage of finding the most efficient driving algorithm. Further studies to increase the driving speed are required for advanced eye-tracking systems, light detection systems, and ranging applications.

## Figures and Tables

**Table 1:** Material parameters of HBM-2 nematic liquid crystal mixture, at room temperature.

Parameter	Quantity for HBM-2 Liquid Crystal
**Dielectric Anisotropy *ε*∥**	16.1
**Dielectric Anisotropy *ε*⊥**	3.55
**Ordinary refractive index *n_o_*** **(*λ* = 460 nm/520 nm/638 nm)**	1.5547/1.5428/1.5292
**Extraordinary refractive index *n_e_*** **(*λ* = 460 nm/520 nm/638 nm)**	1.9198/1.8802/1.8333
**Elastic Constant K_11_ (J/m)**	26 × 10^−12^
**Elastic Constant K_22_ (J/m)**	5 × 10^−12^
**Elastic Constant K_33_ (J/m)**	29 × 10^−12^
**Viscosity (Pa·s)**	0.75

## Data Availability

Data underlying the results presented in this paper are not publicly available at this time but may be obtained from the authors upon reasonable request.
